# Deletion of TLR4 reduces apoptosis and improves histology in a murine kidney transplant model

**DOI:** 10.1038/s41598-021-95504-7

**Published:** 2021-08-10

**Authors:** Swati Jain, Robert Plenter, Trevor Nydam, Ronald G. Gill, Alkesh Jani

**Affiliations:** 1 University of Colorado Denver Division of Renal Diseases and Hypertension, University of Colorado Anschutz Medical Center, 12700 East 19th Avenue, C281, Aurora, CO 80045 USA; 2 Denver Veterans Affairs Medical Center, 1055 Clermont St, Denver, CO 80220 USA

**Keywords:** Nephrology, Kidney

## Abstract

Acute kidney injury (AKI) after transplantation of human deceased donor kidneys is associated with upregulation of tubular toll like receptor 4 (TLR4), but whether TLR4 is required for AKI is unknown. We hypothesized that TLR4 knockout mice (TLR4KO) subjected to cold ischemia followed by kidney transplant (CI + Txp) would be protected from AKI. C57Bl/6J wild type or TLR4KO kidneys were subjected to CI + Txp into wild type recipients. Tubular cell apoptosis, tubular injury and cast formation were significantly improved in recipients of TLR4KO kidneys. TLR4KO kidneys also demonstrated significantly decreased expression of the effector caspase 8. Brush border injury scores and serum creatinine were not different in recipients of TLR4KO versus wild type kidneys. Phosphorylated RIP3 and MLKL through which TLR4 signals programmed necrosis were expressed in both recipient groups. In addition, TNF-α and TNFR1 expression were significantly increased in recipient serum and TLR4KO kidneys respectively after CI + Txp, suggesting continued activation of programmed necrosis despite TLR4 deletion. Our results suggest that TLR4 deletion decreases apoptosis via inhibition of the death receptor pathway and decreases tubular injury and cast formation.

## Introduction

Tubular cell apoptosis is a feature of cold ischemic injury in both human^1–4^ and mouse donor kidneys. We have previously demonstrated in mice that prolonged cold ischemia (CI) is a risk factor for acute kidney injury (AKI) after kidney transplantation^3^. We have also shown that mouse kidneys subjected to cold ischemia followed by transplantation (CI + Txp) have significantly increased tubular apoptosis in association with increased TLR4 and caspase 8 protein expression compared to kidneys transplanted without cold ischemia^5^. Whether TLR4 and caspase 8 are directly responsible for apoptosis seen during cold ischemic injury is unknown.

Acute Tubular Necrosis (ATN) has been traditionally seen as an uncontrolled and passive process^6^. However, recent work suggests that necrosis may occur as a regulated process mediated by receptor-interacting protein kinase 3 (RIP3)^7,8^ in response to Toll-like receptor 4 (TLR4) stimulation^7–10^. In this regard, increased TLR4 expression is seen in human deceased-donor versus living-donor kidneys not exposed to cold ischemia^11^, and donor kidneys with TLR4 loss-of-function mutations have higher rates of immediate graft function versus wild-type kidneys^11^. We have previously shown that donor kidneys subjected to cold ischemia followed by kidney transplantation (CI + Txp) have significantly higher ATN scores in association with increased RIP kinase 3 (RIP3) and TLR4 protein expression^5^. However, whether increased TLR4 is actually required for this regulated, programmed necrosis is unknown. We hypothesized that deletion of TLR4 would protect mouse donor kidneys subjected to cold ischemia followed by kidney transplantation (CI + Txp) from apoptosis and programmed necrosis. We compared, apoptosis, histology and function of donor kidneys from TLR4 knockout (TLR4KO) mice to wild type donor kidneys subjected to cold ischemia followed by kidney transplantation (CI + Txp).

## Methods

### In vitro cold storage–rewarming (CS/REW) model

M-1 (ATCC CRL-2038) renal tubular epithelial cells (RTECs) were subjected to cold storage (CS) in cold saline for 24 h at 4 °C and rewarmed (REW) in normal media at 37 °C for 24 h as previously described^3,12–14^.

### Animals

Inbred male wild-type (WT) C57Bl/6J and TLR4 knockout mice on a C57Bl/6J background (B6(Cg)-Tlr4tm1.2Karp/J, 029015), weighing 20–25 g were purchased from the Jackson Laboratory (Bar Harbor, ME) and were housed under pathogen-free conditions at the University of Colorado Denver, Barbara Davis Center Animal Facility according to NIH Guidelines and with approval of the University of Colorado Denver IACUC. Experiments were performed at the Transplant Microsurgery Facility (University of Colorado). The study protocol was approved by the University of Colorado Denver Institutional Animal Care and Use Committee. The study was carried out in compliance with the ARRIVE guidelines.

### Mouse kidney transplant

Kidney transplants were performed as previously described^15,16^. Briefly, after perfusion with heparinized saline the donor kidney, vessels and ureter from a WT or TLR4KO mouse, were removed and stored in saline at 4 °C for 30 min until transplantation. The WT recipient mice were prepared before the end of the cold storage period. Following bilateral recipient nephrectomy, the donor kidney was placed in the right flank and the arterial cuff was anastomosed to the recipient aorta and the renal vein was anastomosed to the inferior vena cava. The recipient bladder was prepared and the ureter anastomosed to the bladder according the procedure developed by Han et al.^17^ Thus, WT or TLR4KO kidneys subjected to cold storage were transplanted into WT recipients. Total implant time was approximately 30–40 min. Anesthesia was achieved with pentobarbital (60 mg/kg IP), and analgesia was attained with buprenorphine SR (1.0 mg/kg SC).

The transplanted kidney was removed on post-transplant day 1 and serum creatinine was measured as previously described by our group and other groups^3,18^. The kidney transplant was then immediately sectioned for snap freezing in liquid nitrogen, or fixation in 10% phosphate-buffered formalin (cold storage plus transplant group). All animals demonstrated excellent reperfusion at the time of transplant. Similarly, the warm- and cold-ischemia times were not different between the groups.

### Renal function

Serum creatinine was measured using a creatinine enzymatic kit (Pointe Scientific, C7548) on kidney transplant recipients of WT and TLR4KO kidneys on POD1.

### Renal histology

All histological parameters were assessed by a nephropathologist and observers blinded to the treatment modality. Slides were stained for PAS and scanned using the Aperio scanner. Histological injury was quantitated by counting the percent of tubules that displayed loss of brush border, cast formation, tubular simplification and tubule dilatation as previously described^1,3,13,19,20^. Each high-power field (400 ×) was divided into 6 quadrants. Every parameter was examined in both cortex and medulla and at least 150–200 tubules were examined per section.

### Brush border injury score

Tubules that displayed the loss of brush border were quantified as previously described^1,3,13,19,20^. Each tubule was assessed for severity of brush-border injury as follows: circumference of the tubule with less than 25% of BBI was given a score 1; tubule with 26–50% BBI was given a score 2; tubule with 51–75% BBI was given a score of 3; and a tubule with more than 76% of BBI was given a score of 4. The total score was calculated as mean BBI score per number of tubules in each quadrant.

### Cast formation and tubular simplification

Each tubule was checked for cast formation and number of cast were counted in each quadrant^1,3,13,19,20^. Tubular injury was quantified by the presence of flattened tubular cells with nuclei and minimal cytoplasm in the tubule.

### Morphological quantification of apoptosis

Cellular rounding and shrinkage, nuclear chromatin compaction and formation of apoptotic bodies were accounted for the detection of the apoptotic tubular epithelial cells on PAS staining^1,13,19,21^. Apoptotic tubular epithelial cells were quantified in blinded fashion per high-power field (400 ×).

### Apoptotic cell detection via TUNEL staining

Nuclear DNA fragmentation, an important biochemical hallmark of apoptosis was used to detect apoptotic cells using DeadEnd Colorimetric TUNEL assay kit (Promega, G7130). The brown colored TUNEL positive cells were quantified at 400 × magnification in 10 randomly selected quadrant fields in blinded fashion^3,13^.

### Western blot analysis

Western bolt experiments were performed with a primary antibody for TLR4 (Cell Signaling, 2219), Cleaved caspase-8 (Cell Signaling, 8592), RIP3 (ProScience, 2283), phospho RIP3 (Cell Signaling, 57220), MLKL (Santa Cruz Biotech, sc-165025), phospho MLKL (Cell Signaling, 37333), TNFR1 (Santa Cruz Biotech, sc8436) and βactin (cell signaling, 4970/58169) overnight at 4 °C coupled with appropriate secondary antibody as previously described^3^. ECL was used as a method of detection. Chemiluminescence was recorded with an Image Station 440CF and results analyzed with the 1D Image Software (Kodak Digital Science, Rochester, NY).

### TNF-α ELISA

TNF-α concentration in the serum was quantified by ELISA (R&D systems, MTA00B) following the manufacturer's instructions. Quantification measurement was done relative to standard curves.

### Statistics

Statistical analysis was performed with GraphPad Prism 5 software (GraphPad Software, Inc.). Two groups with n = 7 each were analyzed by unpaired two-tailed Student t test. Results are presented as mean ± SEM and *p* < 0.05 was considered significant. *p* = ns refers to a non-significant value.

## Results

**Figure 1:**
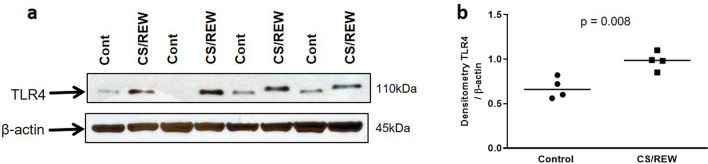
Immunoblot and densitometry for tubular toll like receptor 4 (TLR4) in-vitro: (**a**,**b**) Cells subjected to cold storage followed by rewarming (CS/REW) had significantly increased expression of TLR4 compared to the control cells which were kept at 37 °C (0.980 ± 0.051 vs. 0.675 ± 0.059; **p < 0.01 vs. Control). Immunoblots from three separate experiments were identical to the representative picture. β-actin is used as a protein loading control. Data were analyzed using an unpaired two-tailed Student t test. Values are means ± SEM. n per group = 4. The uncropped blots are presented in Supplementary Figure 1.

We have recently shown that mouse kidneys subjected to cold storage followed by transplantation (CI + Txp) have significantly increased whole kidney expression of TLR4^5^. To determine whether TLR4 is specifically expressed by mouse renal tubular epithelial cells (RTECs), we subjected mouse RTECs to an in-vitro model of cold storage followed by rewarming (CS/REW) as we have previously described^3,12–14^. Mouse RTECs subjected to CS/REW demonstrated a significant increase in expression of TLR4 compared to control cells (Fig. 1).

### Renal histology in kidneys subjected to CI + Txp

**Figure 2:**
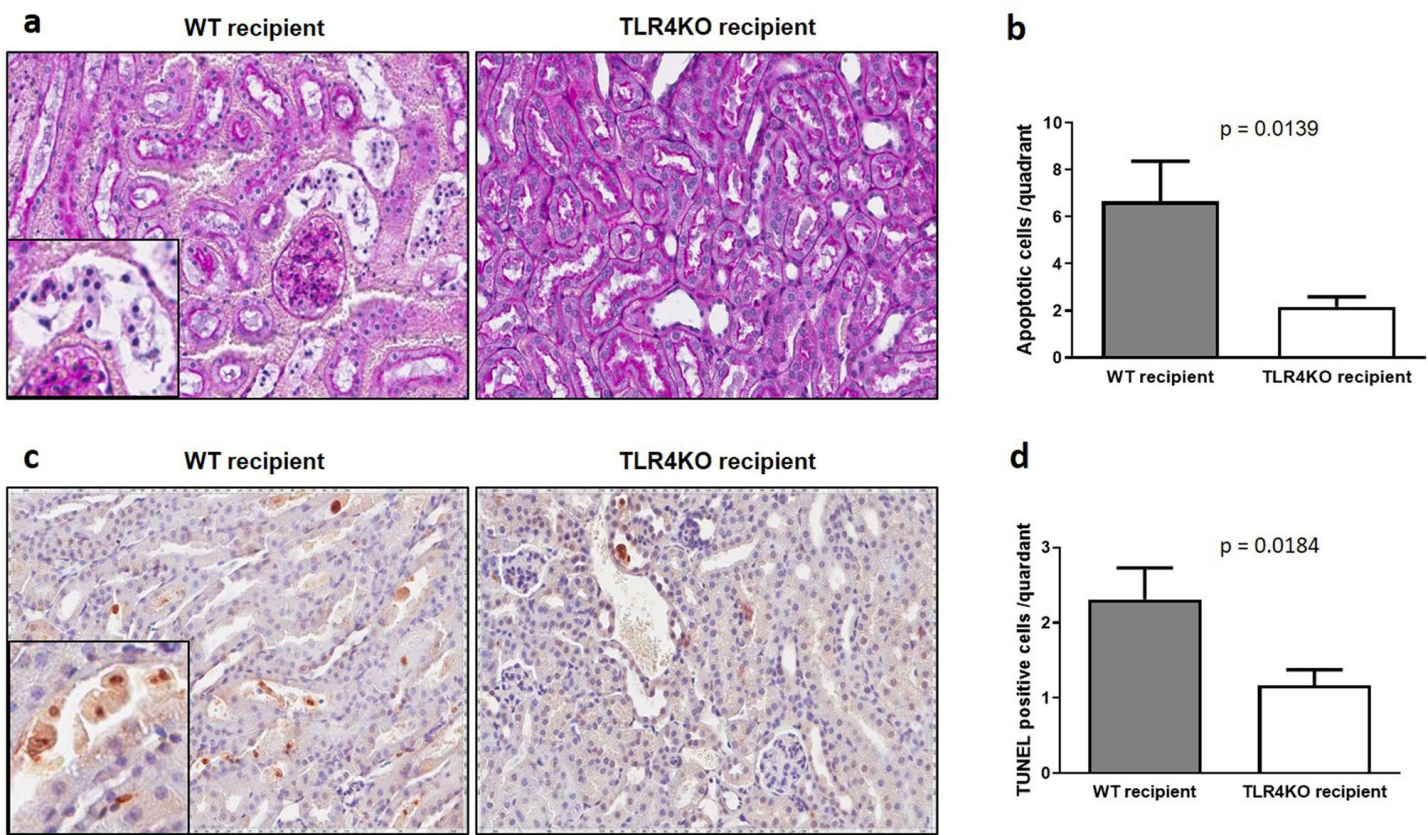
Assessment of tubular cell apoptosis: (**a**) Representative renal histology pictures of transplanted wild type and TLR4 knockout kidneys. Renal tubular apoptotic cells were counted based on morphological criteria (as described in the “Methods” section). (**b**) Kidneys transplanted from TLR4KO donors had significantly fewer apoptotic tubular epithelial cells compared to kidneys transplanted from wild type donors (6.655 ± 1.717 vs. 2.172 ± 0.412; *p < 0.05 vs. TLR4KO-WT). (**c**) Representative pictures of TUNEL staining (brown) demonstrating reduced staining in TLR4KO kidneys and the presence of TUNEL positive epithelial cells in the parenchyma and tubule lumen. (**d**) Kidneys transplanted from TLR4 knockout donors had significantly fewer apoptotic cells compared to kidneys transplanted from wild type donors (2.310 ± 0.421 vs. 1.172 ± 0.205; *p < 0.05 vs. TLR4KO-WT). Data were analyzed using an unpaired two-tailed Student t test. Values are means ± SEM. n per group = 7. WT–WT represents kidney transplanted from wild type donor to wild type recipient and TLR4KO-WT represents kidney transplanted from TLR4 knockout donor to wild type recipient.

We then quantified the number of apoptotic cells both by morphologic assessment and by TUNEL assay. The number of tubular apoptotic cells was significantly reduced in the TLR4KO-WT group versus WT–WT group both morphologically (Fig. 2a,b) and by TUNEL assay (Fig. 2c–d).

**Figure 3:**
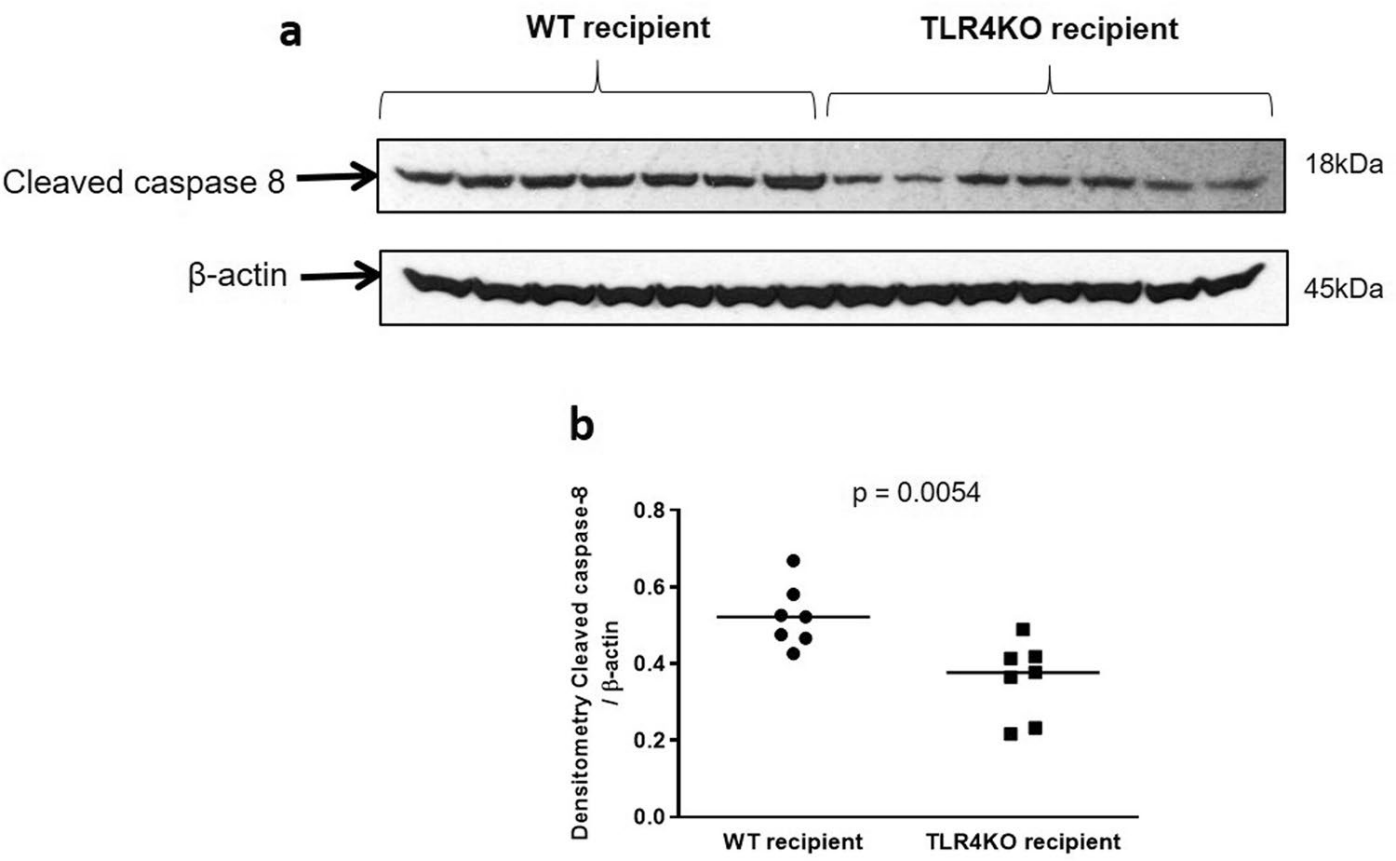
Immunoblot and densitometry for cleaved caspase-8: (**a**,**b**) Transplanted TLR4KO kidneys had significantly less expression of cleaved caspase-8 in comparison to kidneys transplanted from wild type donors (0.532 ± 0.030 vs. 0.358 ± 0.037; **p < 0.01 vs. TLR4KO-WT). β-actin is used as a protein loading control. Data were analyzed using an unpaired two-tailed Student t test. Values are means ± SEM. n per group = 7. WT–WT represents kidney transplanted from wild type donor to wild type recipient and TLR4KO-WT represents kidney transplanted from TLR4 knockout donor to wild type recipient. The uncropped blots are presented in Supplementary Figure 2.

We have previously demonstrated that there is an increase in active caspase-8 expression in kidneys transplanted after cold ischemia^5^. Therefore, we examined the expression of caspase-8 in wild type and TLR4KO kidneys subjected to CI + Txp. Cleaved capsase-8 protein expression was significantly reduced in TLR4 KO kidneys subjected to CI + Txp compared to wild-type (Fig. 3).

**Figure 4:**
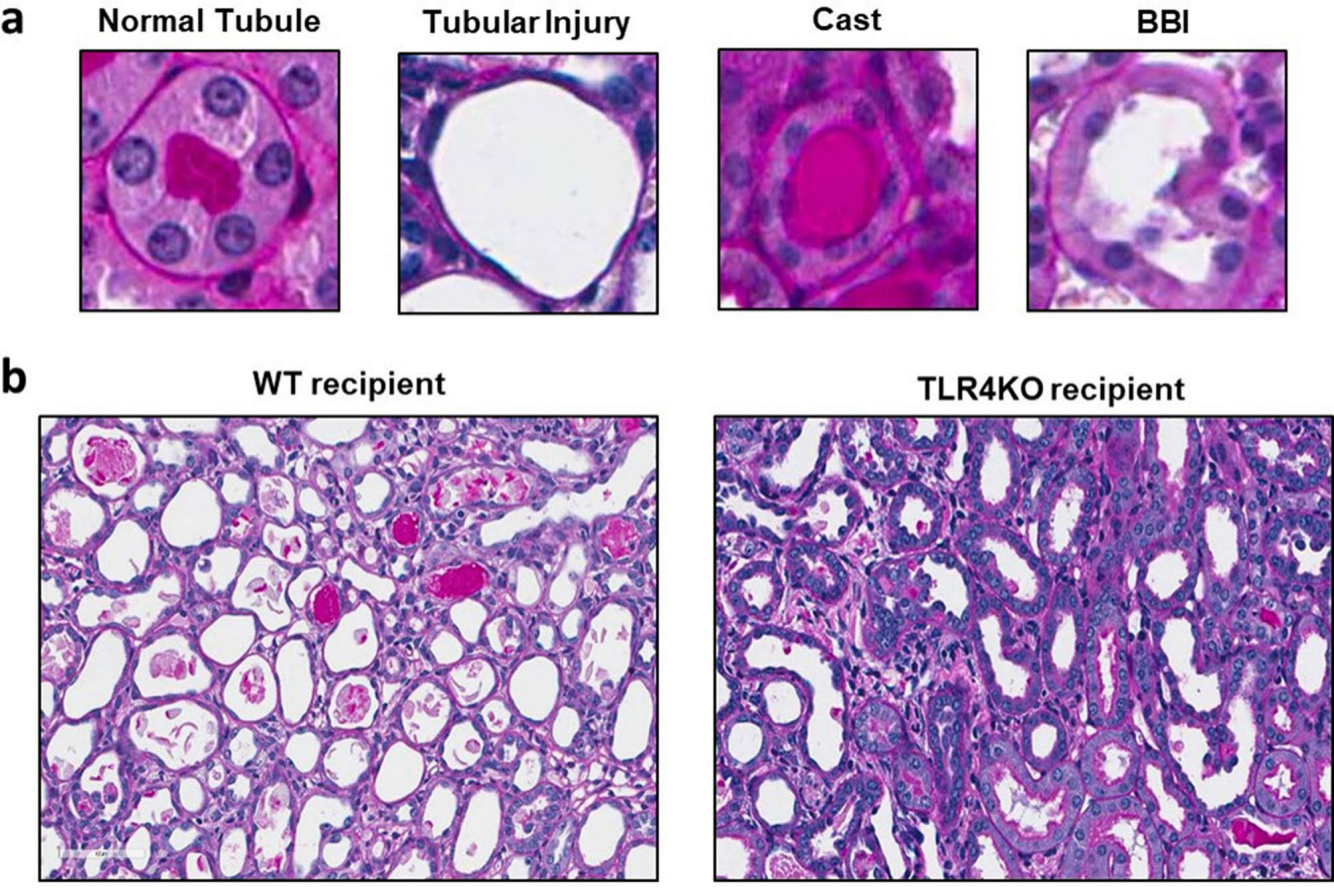
Assessment of tubular injury and cast formation: (**a**) Representative picture of an injured tubule displaying the flattened tubular cells, a cast and a tubule displaying loss of brush border compared to a normal tubule. (**b**) Representative renal histology pictures of transplanted wild type and TLR4 knockout kidneys displaying comparatively less tubular injury and cast formation in TLR4KO kidney recipient compared to WT kidney recipient. However, Brush border injury does not differ much in both the groups.

**Figure 5:**
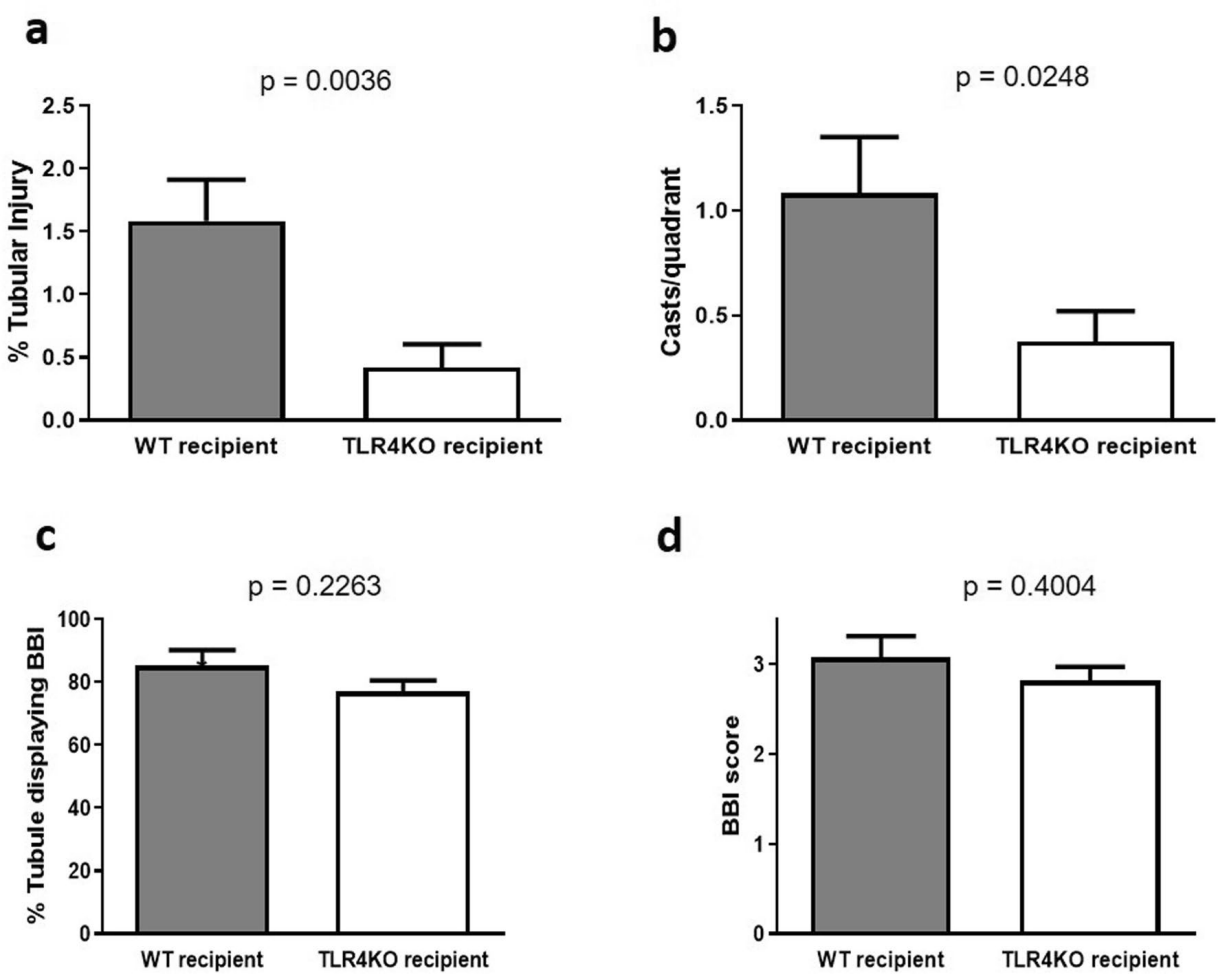
Assessment of brush border injury and its severity: (**a**,**b**) Tubular injury (1.583 ± 0.329 vs. 0.416 ± 0.189) and cast formation (1.083 ± 0.268 vs. 0.375 ± 0.145) were significantly reduced in kidneys transplanted from TLR4 knockout donors compared to kidneys transplanted from wild type donors (**p < 0.01 vs. TLR4KO-WT for tubular injury and *p < 0.05 vs. TLR4KO-WT for cast formation). (**c**,**d**) The percent of tubules displaying Brush-border injury (85.25 ± 5.0 vs. 76.98 ± 3.55) and its severity (3.075 ± 0.237 vs. 2.820 ± 0.151) based on the percent of the circumference of the tubule displaying BBI (please see “Methods” section) both did not change significantly in transplanted wild type and TLR4 knockout kidneys (*p* = ns), *ns* not significant. Data were analyzed using an unpaired two-tailed Student t test. Values are means ± SEM. n per group = 7. WT–WT represents kidney transplanted from wild type donor to wild type recipient and TLR4KO-WT represents kidney transplanted from TLR4 knockout donor to wild type recipient.

Histological parameters (Fig. 4a) were also assessed in both control and TLR4KO groups. Histological examination also revealed that wild type kidneys subjected to CI and then transplanted into WT recipients had significant loss of tubular cell volume and tubular simplification quantified as tubular injury (Figs. 4b, 5a) and cast formation (Figs. 4b, 5b). In contrast, TLR4KO kidneys subjected to CI and then transplanted had significantly less tubular injury and cast formation (Figs. 4, 5).

Brush border injury and its severity were not significantly different between TLR4KO and WT donor kidneys subjected to CI + Txp (Figs. 4b, 5c,d).

### Renal function

**Figure 6:**
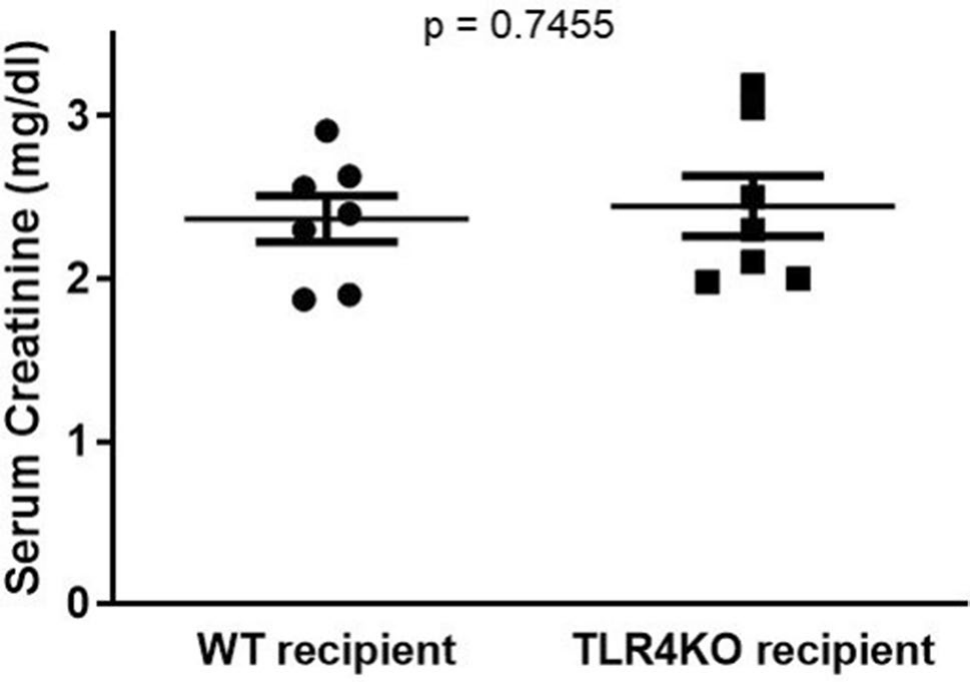
Measurement of kidney function: Recipient serum creatinine did not differ significantly after transplantation of TLR4KO donor kidneys (TLR4KO-WT) compared to wild type donor kidneys (WT–WT) (2.367 ± 0.144 vs. 2.446 ± 0.187; *p* = ns), ns = not significant. n per group = 7. Data were analyzed using an unpaired two-tailed Student t test. Values are means ± SEM. n per group = 7. WT–WT represents kidney transplanted from wild type donor to wild type recipient and TLR4KO-WT represents kidney transplanted from TLR4 knockout donor to wild type recipient.

Interestingly, despite differences in several of the features described above between wild type and TLR4KO kidneys, kidney function as assessed by serum creatinine was not significantly different in the recipients of WT or TLR4KO kidneys (WT–WT sCr = 2.49 ± 0.22 vs. TLR4KO-WT sCr = 2.71 ± 0.25) (Fig. 6).

### Effectors of programmed necrosis

**Figure 7:**
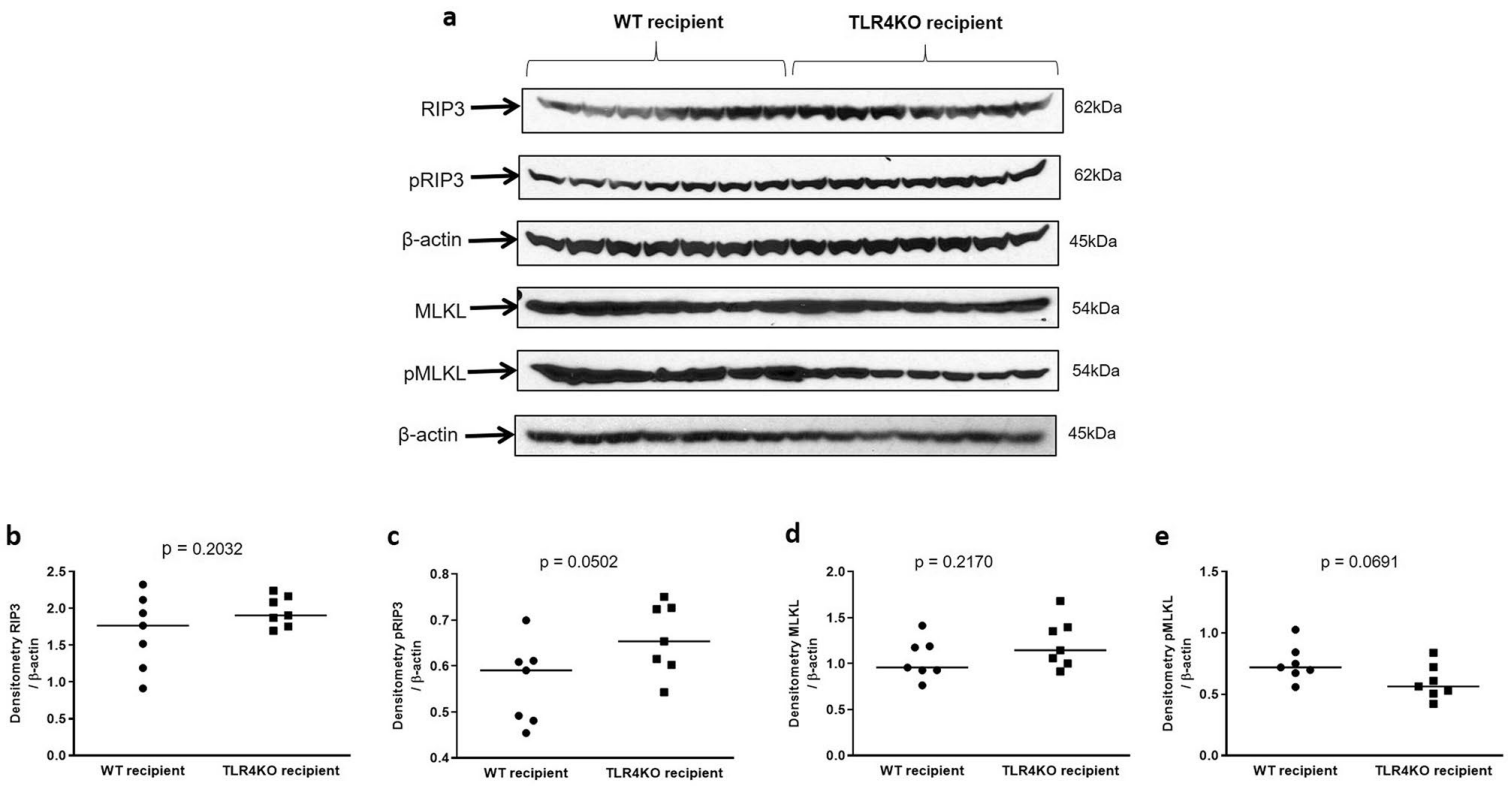
Immunoblot and densitometry for Receptor-interacting serine/threonine-protein kinase 3 (RIP3) and Mixed lineage kinase domain-like protein (MLKL): (**a**–**e**) Protein expression of RIP3 (1.682 ± 0.191 vs. 1.96 ± 0.078; *p* = ns) and its phosphorylated form at Thr231/Ser232 (0.562 ± 0.033 vs. 0.659 ± 0.029; *p* = ns), as well as MLKL (1.051 ± 0.082 vs.1.222 ± 0.0101; *p* = ns) and its phosphorylated form, Ser345 (0.752 ± 0.055 vs. 0.598 ± 0.053; *p* = ns) were not significantly different in transplanted wild type and TLR4 knockout kidneys. β-actin is used as a protein loading control. Data were analyzed using an unpaired two-tailed Student t test. Values are means ± SEM. n per group = 7. WT–WT represents kidney transplanted from wild type donor to wild type recipient and TLR4KO-WT represents kidney transplanted from TLR4 knockout donor to wild type recipient. The uncropped blots are presented in Supplementary Figure 3.

In addition, we examined WT and TLR4KO kidneys for the presence of effectors of programmed necrosis, namely receptor interacting protein kinase 3 (RIP3), and its substrate, mixed lineage kinase domain like protein (MLKL)^7^. Total and phospho RIP3 as well as MLKL and its phosphorylated form (pMLKL) were not significantly different in recipients of wild type donor kidney or TLR4 knock out kidneys (Fig. 7a–e) suggesting continued activation of programmed necrosis despite TLR4 deletion.

**Figure 8:**
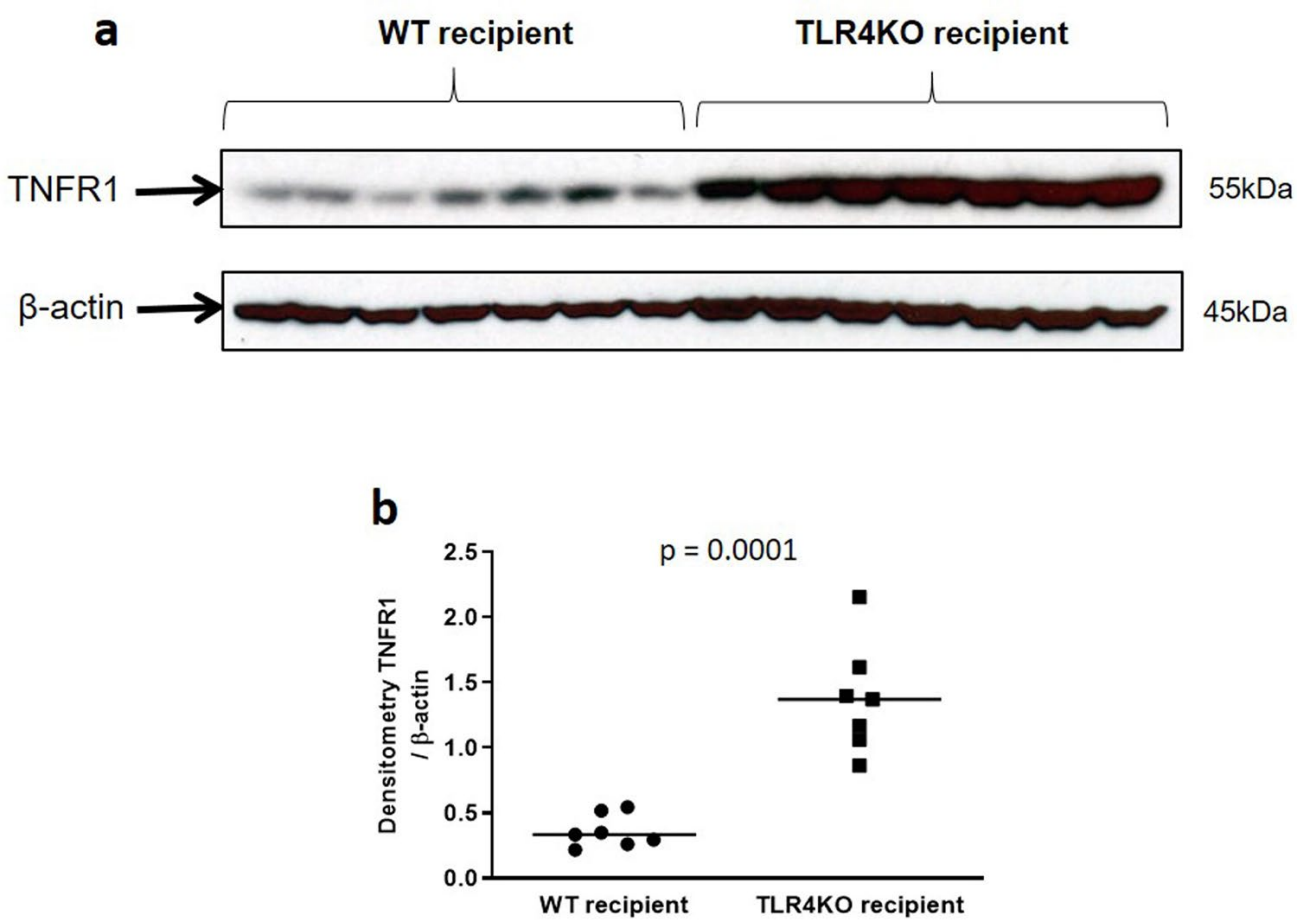
Immunoblot and densitometry for Tumor necrosis factor receptor 1 (TNFR1): (**a**,**b**) Kidneys transplanted from TLR4 knockout donors had significantly increased expression of TNFR1 compared to kidneys transplanted from wild type donors (0.360 ± 0.047 vs. 1.377 ± 0.159; ****p < 0.0001 vs. WT–WT). β-actin is used as a protein loading control. Data were analyzed using unpaired two-tailed Student t test. Values are means ± SEM. n per group = 7. WT–WT represents kidney transplanted from wild type donor to wild type recipient and TLR4KO-WT represents kidney transplanted from TLR4 knockout donor to wild type recipient. The uncropped blots are presented in Supplementary Figure 4.

**Figure 9:**
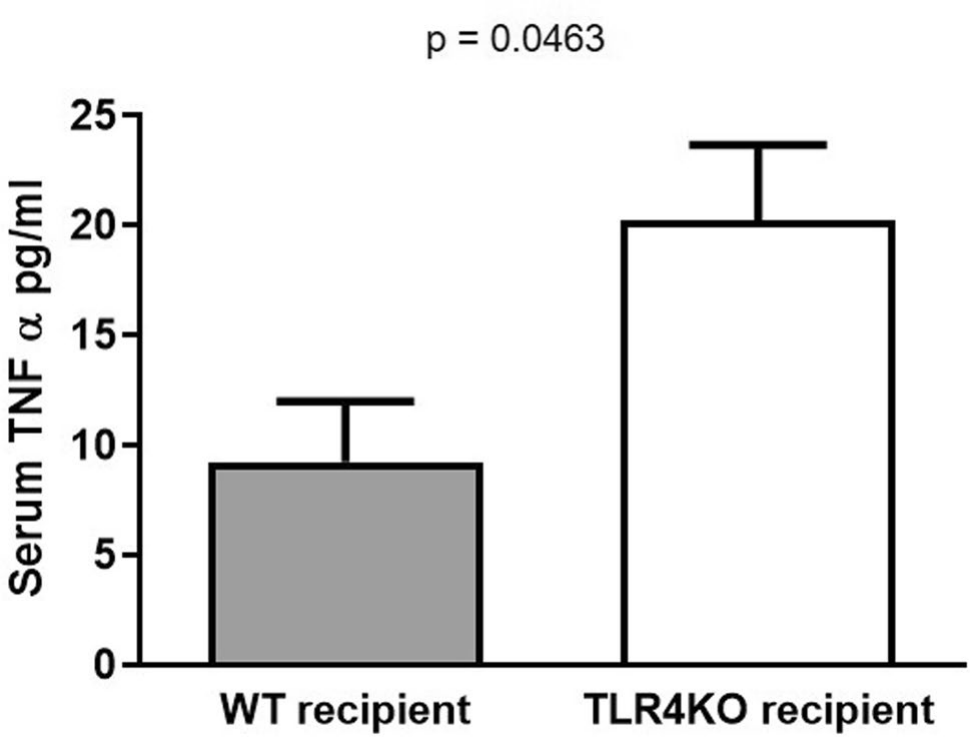
Serum Tumor necrosis factor-alpha (TNF-α) levels: Recipients of TLR4KO donor kidneys have increased serum TNF-α compared to recipients of wild type donor kidneys (9.250 ± 2.750 vs. 20.25 ± 3.425; *p < 0.05 vs. WT–WT). n per group = 7. Data were analyzed using unpaired two-tailed Student t test. Values are means ± SEM. n per group = 7. WT–WT represents kidney transplanted from wild type donor to wild type recipient and TLR4KO-WT represents kidney transplanted from TLR4 knockout donor to wild type recipient.

Since necroptosis is also mediated by death receptors^22^ we then looked for expression of TNFR1 and its ligand TNF-α^23,24^. Both TNFR1 and serum TNF-α were significantly, increased in TLR4KO kidneys and recipient serum respectively after CI + Txp compared to WT kidneys (Figs. 8, 9), suggesting another possible pathway to trigger necroptosis.

## Discussion

Toll-like receptors (TLRs) are an evolutionarily conserved family of pattern recognition receptors that recognize microbial molecules and activate the innate immune system^25^. Toll-like receptors are also present on innate immune cells and on T and B lymphocytes and have been proposed as a bridge between the innate and acquired immune systems^26^.

TLR4 is constitutively expressed on tubular cells and is important in mediating the inflammatory response to bacterial products^25^, diabetes^27^ and warm ischemia reperfusion injury^28^. Moreover, TLR4 is upregulated during renal injury due to inflammation from ischemia or nephrotoxic drugs^26^.

In the context of kidney transplantation, AKI after transplantation of human deceased donor kidneys is associated with upregulation of TLR4^11^, but the mechanism and causal connection by which TLR4 may mediate AKI is unknown. An association has been demonstrated between immediate graft function and two co-segregating missense TLR4 mutants, Asp299Gly and Thr399Ile. Immediate graft function was significantly higher in recipients of a donor kidney with a mutated TLR4. The human studies highlight an important association between TLR4 and AKI after transplantation but the mechanism by which protection is mediated by TLR4 mutations is unclear.

We have previously demonstrated that renal tubular epithelial cell apoptosis is increased by in vitro cold storage followed by rewarming^13,21^. Furthermore, we have recently shown that murine donor kidneys subjected to cold ischemia followed by transplantation (CI + Txp) have significantly more tubular cell apoptosis and ATN than wild type control kidneys, and kidneys subjected to either cold ischemia alone or transplantation alone^5,13,21^. The latter occurred in the setting of increased whole kidney TLR4 and caspase-8 protein expression. TLR4 is associated with activation of caspase 8 and the extrinsic apoptotic pathway^29–31^. It is possible therefore that TLR4 mediates injury by increasing apoptosis, necrosis or both during CI + Txp.

In the current study we first confirmed that TLR4 can be expressed on tubular epithelial cells and is upregulated by cold storage and rewarming (Fig. 1). Next, we found that tubular cell apoptosis was significantly reduced in recipients of TLR4KO kidneys compared to recipients of WT kidneys. We have also previously shown that CI + Txp primarily induces the extrinsic pathway of apoptosis, along with increased expression of TLR4^5^. In contrast transplanted donors kidneys without cold ischemia (i.e., with warm ischemia/reperfusion alone) had excellent renal function, low caspase 8 protein expression which was not significantly different to non-transplanted controls, and little apoptosis which was not significantly different to non-transplanted controls^5^.

In the current study we observed a significant decrease in the protein expression of caspase 8 in TLR4KO kidneys suggesting that TLR4 mediates apoptosis after CI + Txp by increasing death receptor pathway apoptosis.

TLR4 has been shown to cause programmed necrosis by signaling through receptor-interacting kinases 1 and 3^7–10^. Phosphorylation of RIP3 at Thr231 and Ser232 leads to activation and homotrimerization of mixed lineage kinase domain-like protein (MLKL)^32,33^, which then anchors to the plasma membrane and triggers programmed necrosis. We have shown that CI followed by transplantation is associated with a significant increase in protein expression of TLR4, RIP1, RIP3 and phospho MLKL (pMLKL)^5^. RIP kinases 1 and 3 can also be activated by TNF-α /TNFR1 signaling^34^ (supplement Figure 5). We found that donor kidney TNFR1 protein expression and recipient serum TNF-α concentration were also significantly increased following transplantation of kidneys subjected to CI^5^. Thus, involvement of TLR4 and TNFR1 in donor kidneys subjected to cold ischemia followed by transplantation implicate the innate immune system in the development of tubular injury and necrosis^26^.

In both the aforementioned human and murine studies, an unanswered question is whether increased TLR4 expression is a cause or simply consequence of injury that occurs after CI + Txp?

In the current study we observed a significant decrease in tubular injury and cast formation in recipients of TLR4KO kidneys compared to recipients of WT kidneys, suggesting a causative role for TLR4 in causing tubular injury after CI + Txp. To our knowledge, TLR4 has not been implicated in the formation renal casts. TLR4 staining of tubular casts was observed in an experimental nephron reduction model of CKD, possibly due to shedding of TLR4 expressing injured tubular cells^35^. Also, TLRs can activate the inflammasome-caspase-1 pathway through caspase-8^36,37^ and we observed less active caspase-8 in TLR4KO kidneys after transplantation. It is possible therefore, that the significantly decreased caspase-8 contributed to decreased tubular cell injury observed in the recipients of TLR4KO kidneys which in turn contributed to decreased cast formation.

Serum creatinine and brush border injury scores were not significantly different in recipients of WT or TLR4KO kidneys. In addition, even though tubular cell apoptosis and injury were significantly reduced, they were not eliminated in TLR4KO kidneys. We previously demonstrated that tubular cell apoptosis and programmed necrosis occurred after CI + Txp in association with increased donor kidney serum TNF-α and donor kidney TNFR1 expression^5^. We therefore examined the recipient serum for the evidence of TNF-α and donor kidneys for evidence of TNFR1 expression and found them to be significantly increased, in association with phospho RIP3 and phospho-MLKL expression. Taken together these findings suggest multiple, possibly redundant pathways exist to trigger programmed necrosis after CI + Txp. In this regard, we previously demonstrated that pan-caspase inhibition of wild-type kidneys subjected to CI + Txp significantly reduced but did not completely eliminate tubular cell apoptosis, again suggesting caspase-independent pathways may play a role in AKI after kidney transplantation^3^. Therefore, a combined approach with inhibition of both TLR4 and TNFR1, as well as caspase dependent and independent apoptosis may be required to completely block apoptosis and programmed necrosis after cold ischemia followed by kidney transplantation.

There are several limitations to our study that should be considered. Due to the sensitivity of mouse kidneys to cold ischemia, we employed a CI time of 30 min, which is shorter than the typical cold ischemia times used for human kidneys. We and other investigators have demonstrated that mouse kidneys subjected to one hour of cold storage had widespread tubular necrosis in ~ 60% of tubules, and increased serum creatinine after transplantation^3,38^. Mouse kidneys have greater susceptibility to cold ischemia than human kidneys, a finding that is consistent with the observation that mouse kidneys are also more susceptible to warm ischemia–reperfusion injury (IRI). Brief periods of ~ 18–30 min of warm IRI result in widespread tubular necrosis and AKI^39–45^. Despite this difference, the shorter periods of cold ischemia in mice still produce histological injury and AKI that closely resembles biopsies of human kidney transplants with DGF^46,47^.

Another limitation of our study was that we did not use a preservation solution such as University of Wisconsin (UW) solution. Our goal was to isolate the role of TLR4 as an independent variable in post-transplant kidney injury. We therefore excluded potentially beneficial interventions such as use of UW solution in order to prevent any confounding protective effect by the preservation solution.

In summary, we have shown that TLR4KO kidneys subjected to CI + Txp have improved histology compared to wild-type kidneys. Both WT and TLR4KO kidneys demonstrated phospho-RIP3 and phospho-MLKL expression in association with increased recipient serum TNF-α and donor kidney TNFR1 expression, suggesting the continued activation of programmed necrosis despite TLR4 deletion. Our results suggest inhibition of multiple pathways will be necessary to completely eliminate apoptosis and necrosis after cold ischemia followed by transplantation (CI + Txp).

## Supplementary Information

Supplementary Information.
