# Supplementary material for: Deletion of TLR4 reduces apoptosis and improves histology in a murine kidney transplant model
Source: Sci Rep. 2021 Aug 10;11:16182. doi: 10.1038/s41598-021-95504-7 (PMC8355104; doi:10.1038/s41598-021-95504-7)
Supplement: Supplementary file 1 — Supplementary Information. [file 41598_2021_95504_MOESM1_ESM.docx]

**Supplement Figure 1:**

**
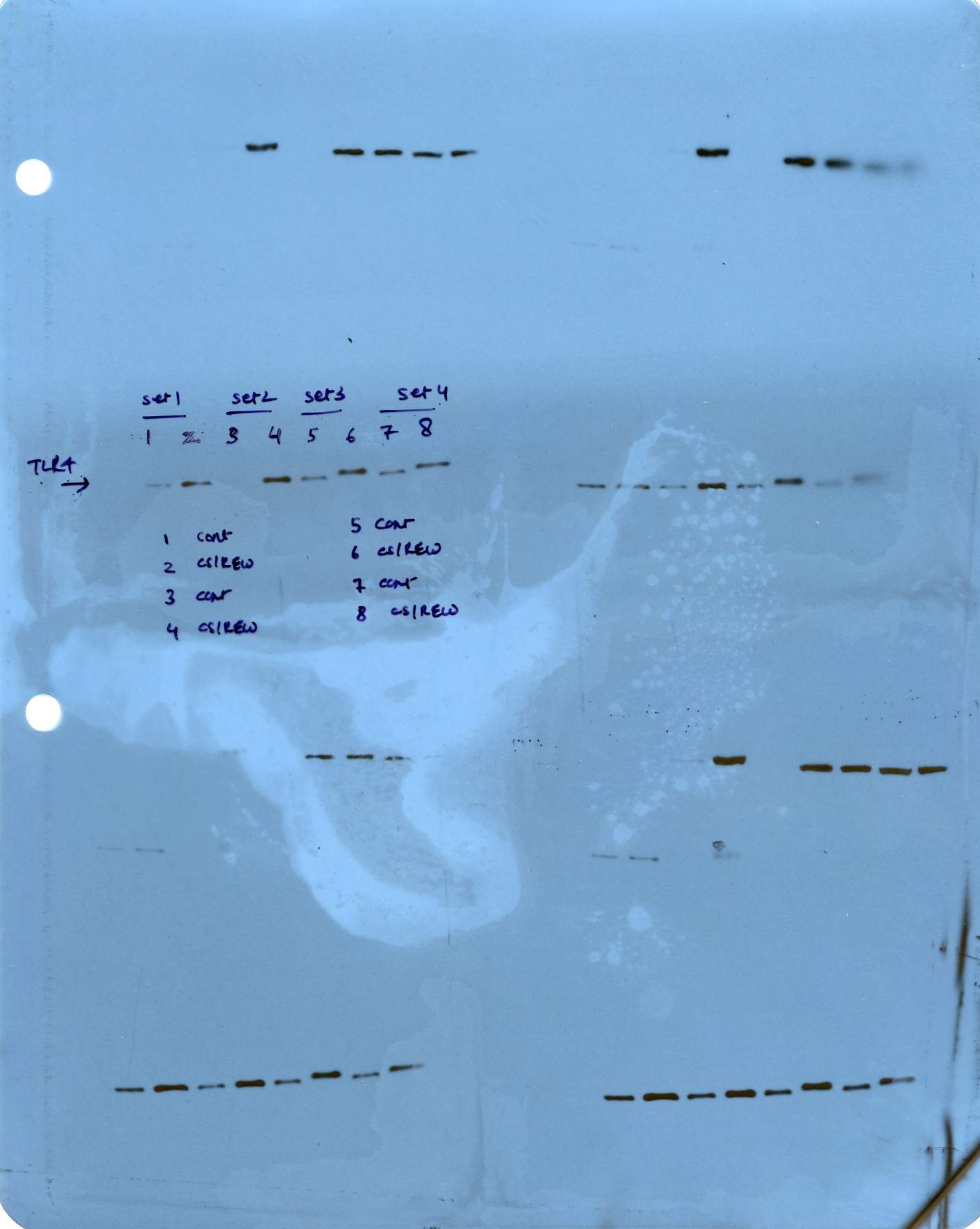
**

**
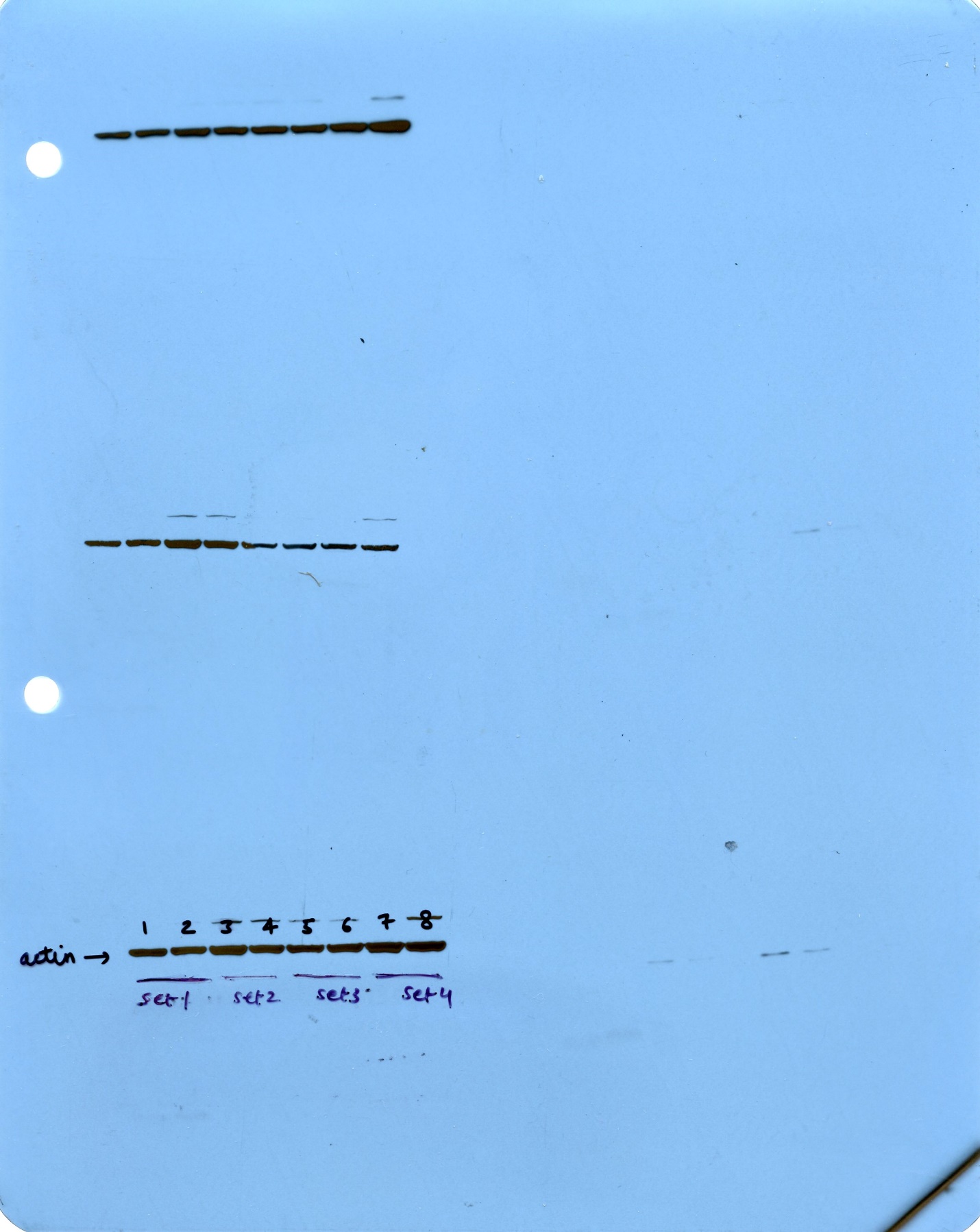
**

**Supplement Figure 2:**

**
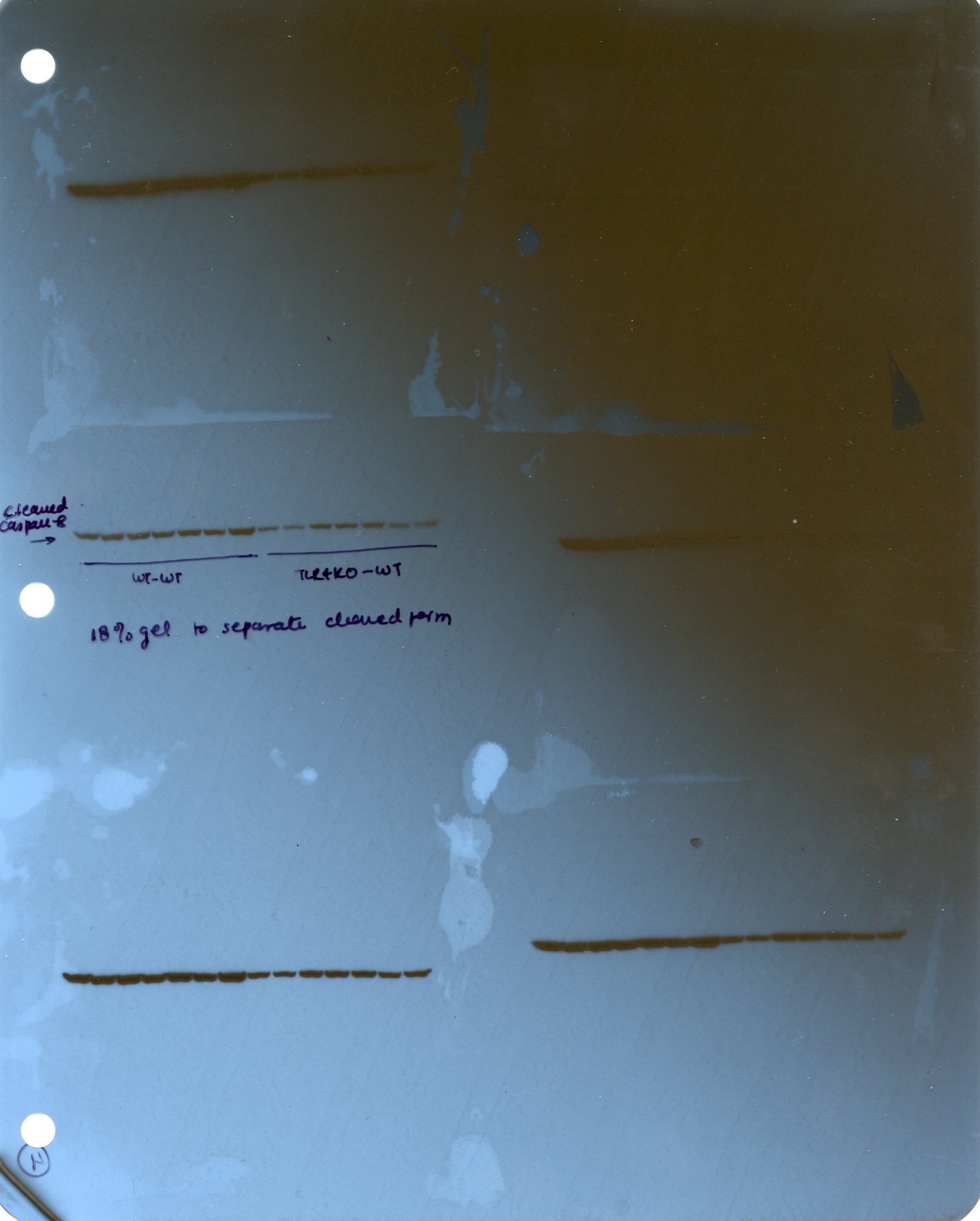
**

**
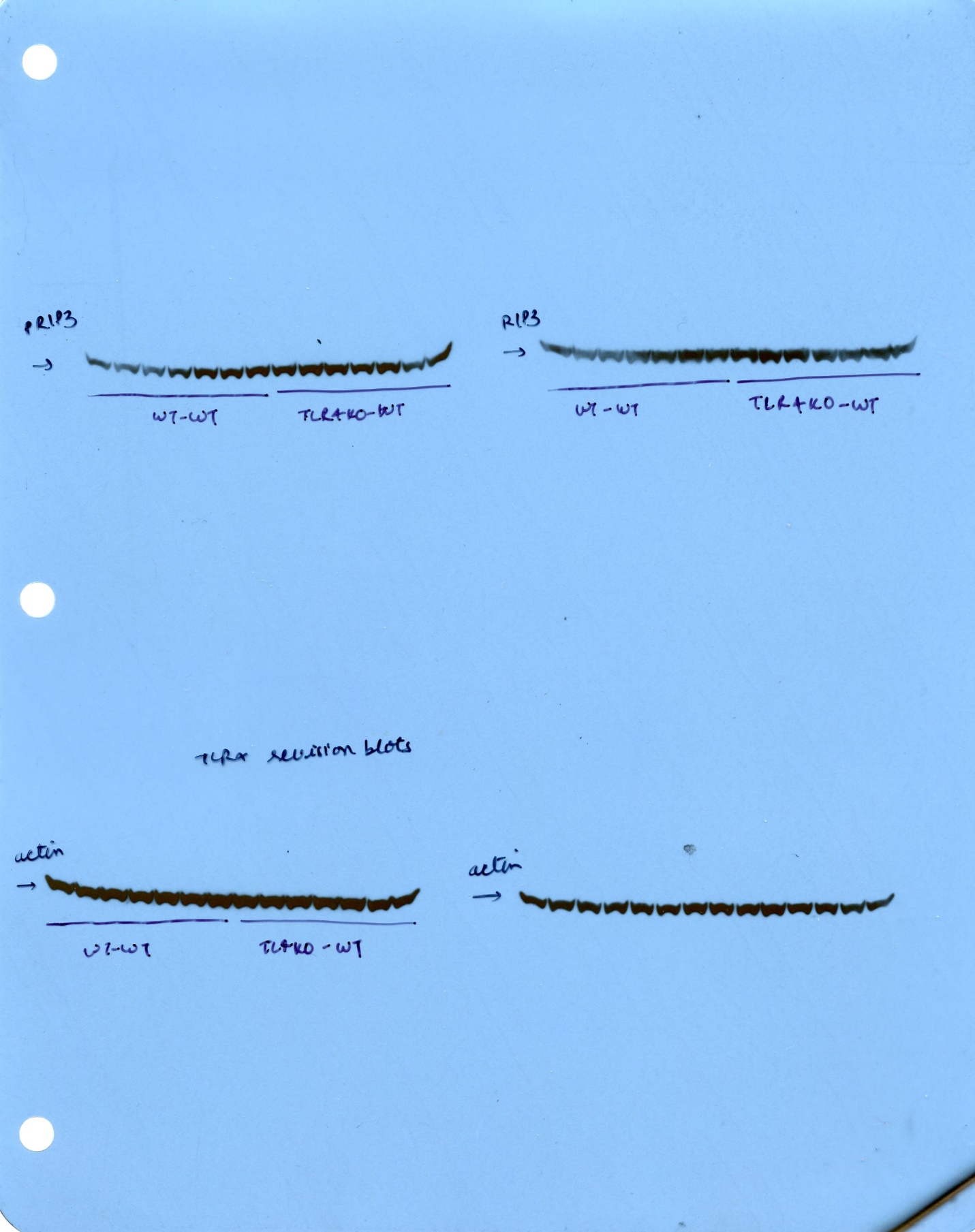
**

**Supplement Figure 3:**

**
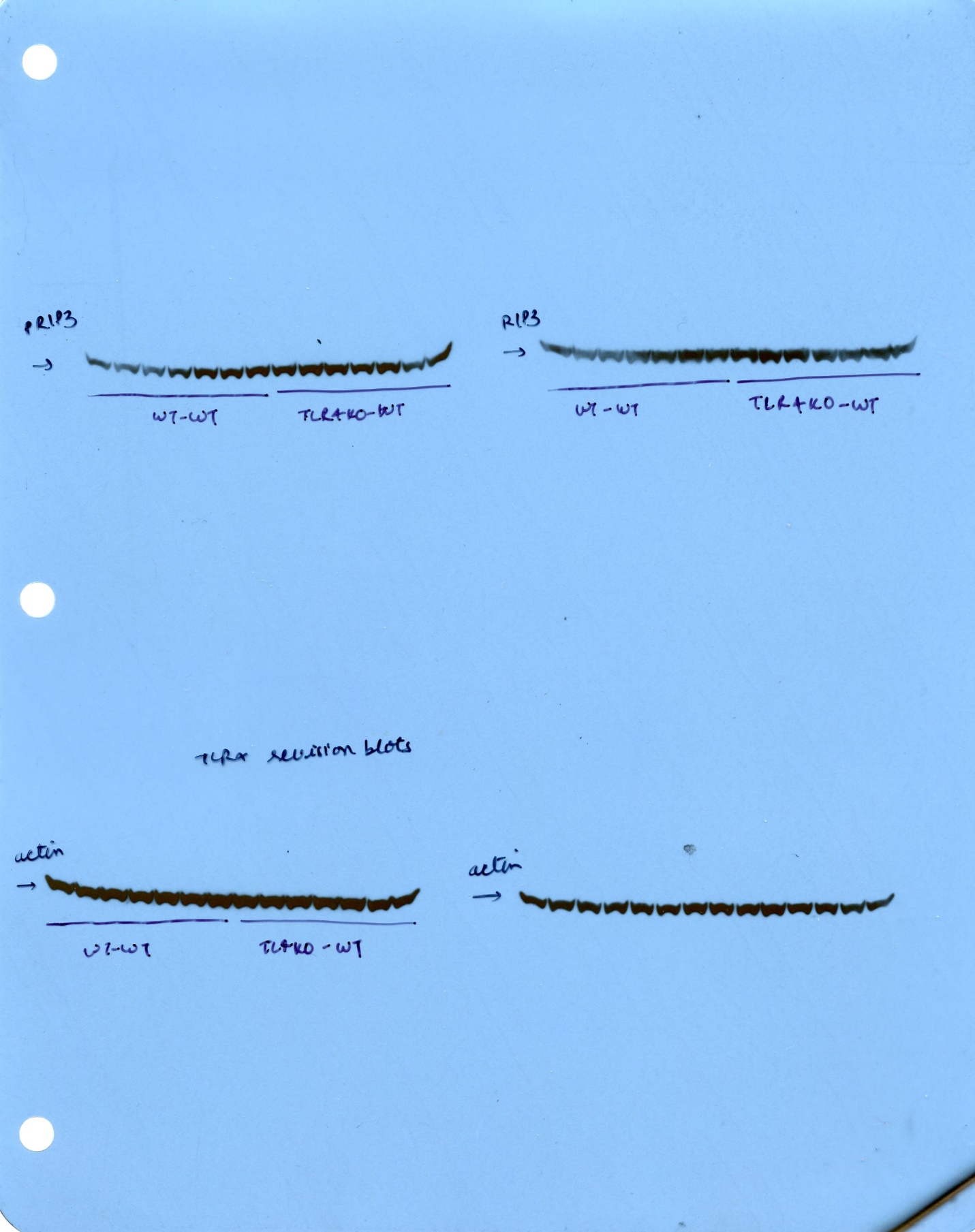
**

**
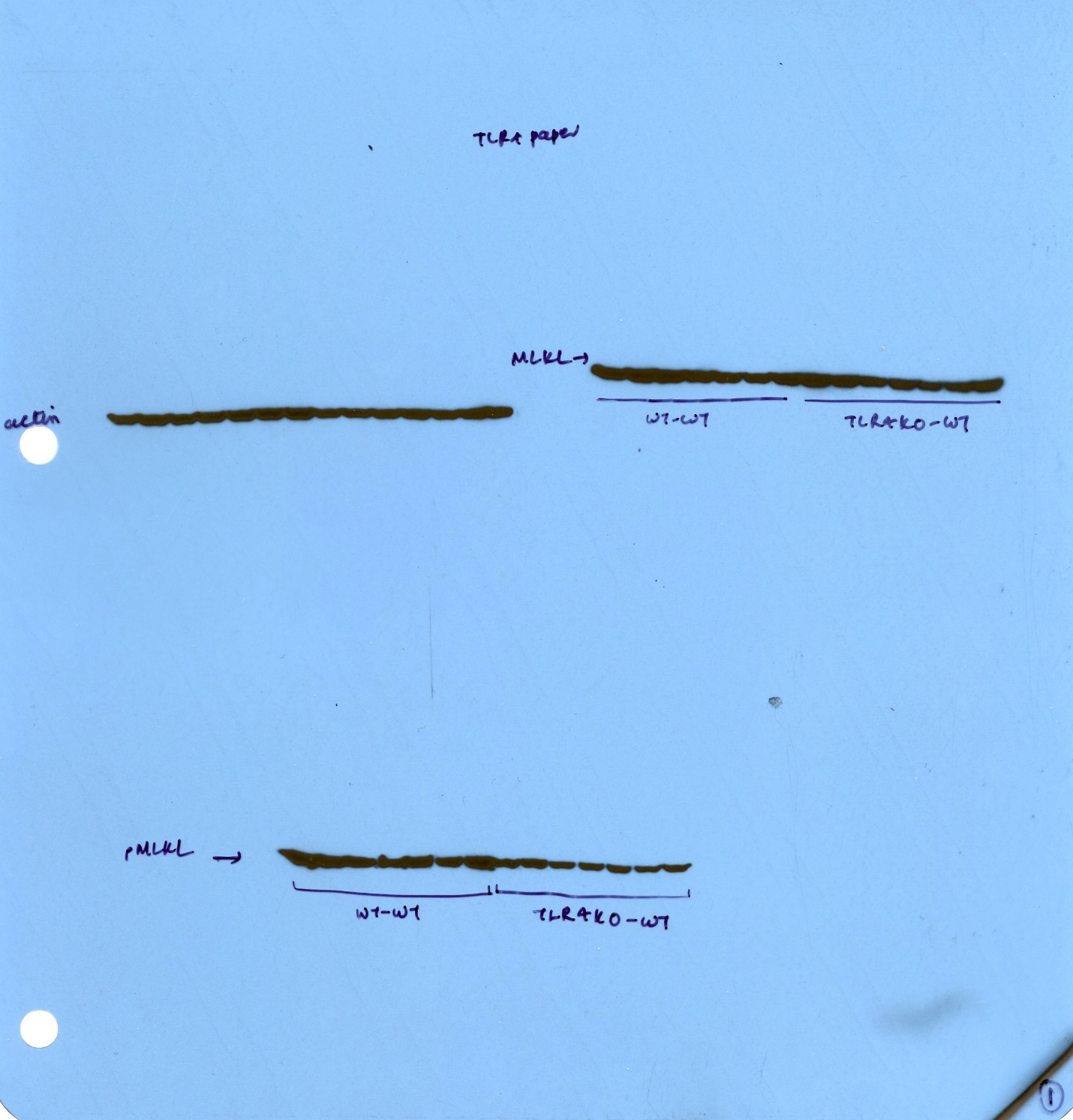
**

**
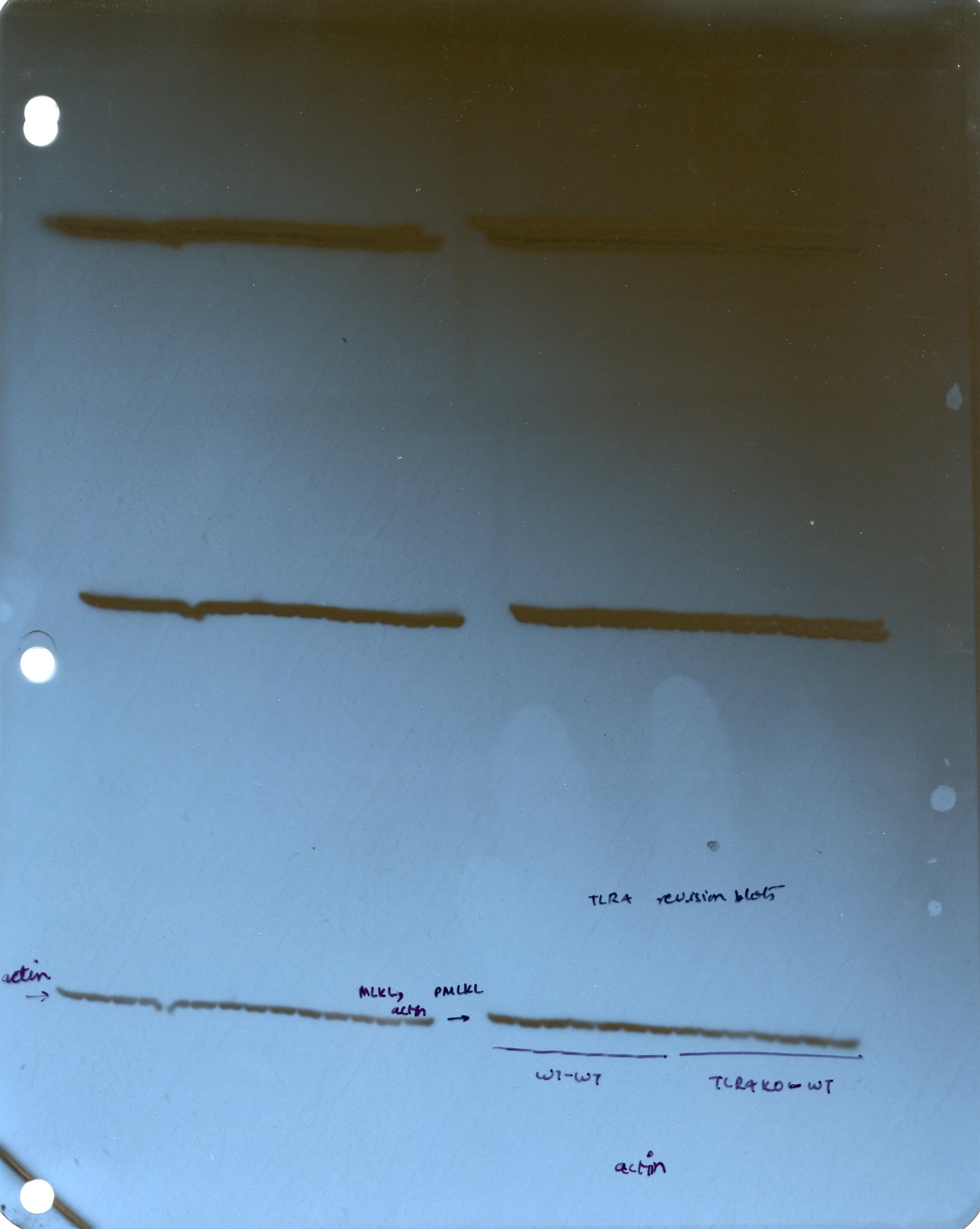
**

**Supplement Figure 4:**

**
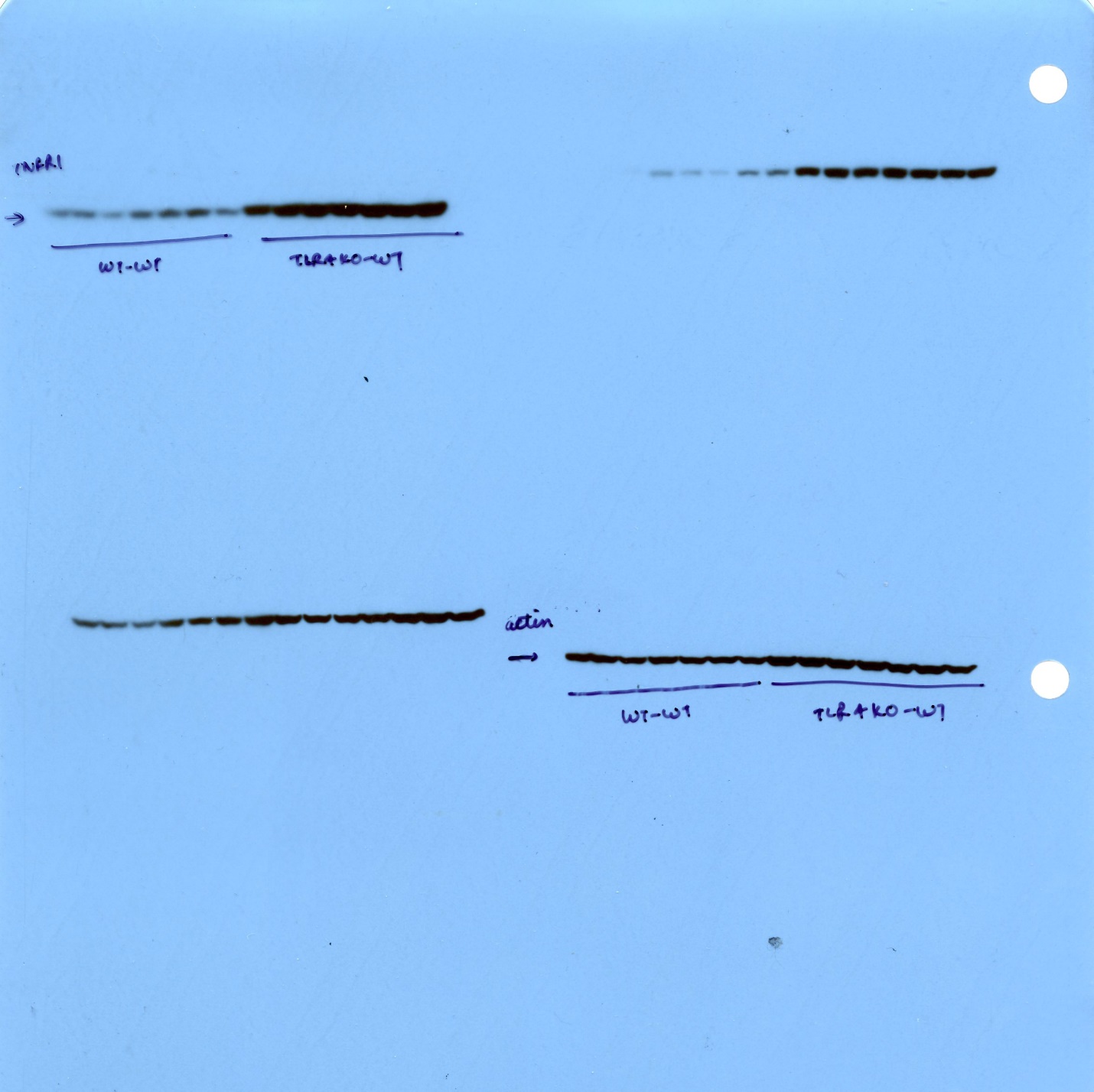
**

**Supplement Figure 5**

**
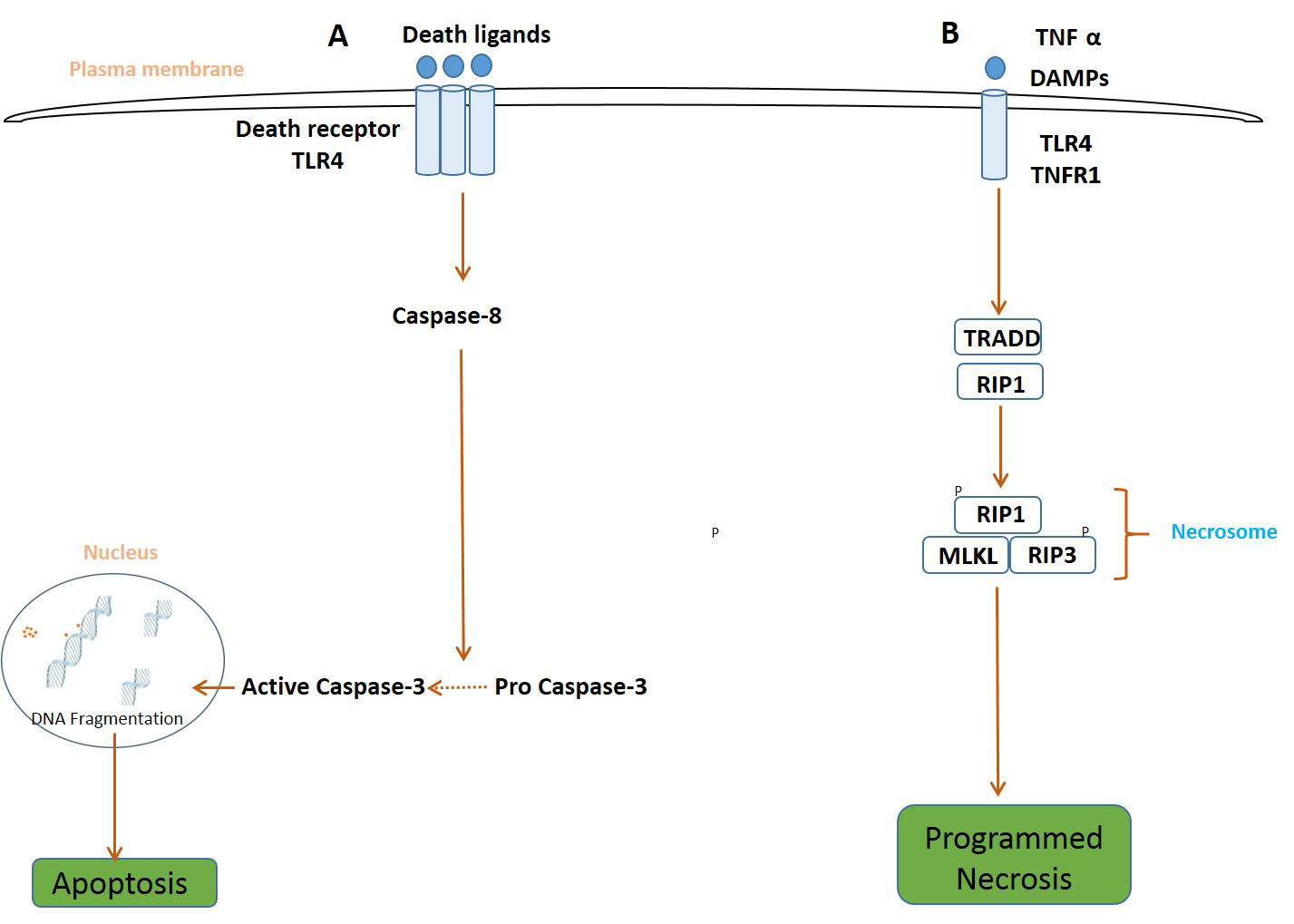
**

**Supplement Figure 5:** Schematic figure illustrating the proposed pathways by which apoptosis and programmed necrosis occur in a donor kidney subjected to cold ischemia followed by kidney transplantation. **(A) Apoptosis**: We have previously demonstrated that donor kidneys subjected to cold ischemia followed by transplantation demonstrate increased protein expression of caspase 8 and TLR4, and increased tubular cell apoptosis ^1^. TLR4 is associated with activation of caspase 8 which in turn cleaves pro-caspase-3 to form active caspase 3, thereby initiating DNA fragmentation and apoptosis ^2,3^. In the current study, TLR4 knockout kidneys had decreased expression of caspase 8 and decreased tubular cell apoptosis (Figures 2 and 3). **(B) Programmed necrosis**: We have also previously shown that donor kidneys subjected to cold ischemia followed by transplantation demonstrate increased protein expression of the effectors of programmed necrosis, including RIP kinase 3 and phospho-MLKL^1^. Toll-like receptor (TLR4) and TNFR1 can activate programmed necrosis through receptor-interacting protein kinase 3 (RIP-3). RIP3 then forms a multi-protein complex known as necrosome with RIP1 and mixed lineage kinase domain like pseudokinase (MLKL). MLKL is phosphorylated in the necrosome and phospho-MLKL translocates to the inner leaflet of the plasma membrane and disturbs the integrity of the cell ^4,5^. In the current study, deletion of TLR4 did not prevent programmed necrosis, possibly due to increased recipient serum TNF-α and donor kidney TNFR1 expression, leading to continued activation of the programmed necrosis pathway.

1 Jain, S., Plenter, R., Nydam, T. & Jani, A. Injury Pathways That Lead to AKI in a Mouse Kidney Transplant Model. *Transplantation* **104**, 1832-1841, doi:10.1097/TP.0000000000003127 (2020).

2 Thornberry, N. A. & Lazebnik, Y. Caspases: enemies within. *Science* **281**, 1312-1316, doi:10.1126/science.281.5381.1312 (1998).

3 Cryns, V. & Yuan, J. Proteases to die for. *Genes & development* **12**, 1551-1570, doi:10.1101/gad.12.11.1551 (1998).

4 Wang, H. *et al.* Mixed lineage kinase domain-like protein MLKL causes necrotic membrane disruption upon phosphorylation by RIP3. *Mol Cell* **54**, 133-146, doi:10.1016/j.molcel.2014.03.003 (2014).

5 Su, L. *et al.* A plug release mechanism for membrane permeation by MLKL. *Structure* **22**, 1489-1500, doi:10.1016/j.str.2014.07.014 (2014).
